# Understanding the local context and its possible influences on shaping, implementing and running social accountability initiatives for maternal health services in rural Democratic Republic of the Congo: a contextual factor analysis

**DOI:** 10.1186/s12913-016-1895-3

**Published:** 2016-11-09

**Authors:** Eric M. Mafuta, Lisanne Hogema, Thérèse N. M. Mambu, Pontien B. Kiyimbi, Berthys P. Indebe, Patrick K. Kayembe, Tjard De Cock Buning, Marjolein A. Dieleman

**Affiliations:** 1 Kinshasa School of Public Health, Faculty of Medicine, University of Kinshasa, PO Box: 11850, Kinshasa I, Democratic Republic of the Congo; 2 Athena Institute, Faculty of Life Sciences, VU University Amsterdam, Amsterdam, The Netherlands; 3 Kongo Central Health Province Division, Muanda, Democratic Republic of the Congo; 4 Agence d’Achat de performances, Muanda, Kongo Central Democratic Republic of the Congo; 5 Royal Tropical Institute, Amsterdam, The Netherlands

**Keywords:** Context analysis, Community participation, Social accountability, DRC, Health committee, Community groups

## Abstract

**Background:**

Social accountability has to be configured according to the context in which it operates. This paper aimed to identify local contextual factors in two health zones in the Democratic Republic of the Congo and discuss their possible influences on shaping, implementing and running social accountability initiatives.

**Methods:**

Data on local socio-cultural characteristics, the governance context, and socio-economic conditions related to social accountability enabling factors were collected in the two health zones using semi-structured interviews and document reviews, and were analyzed using thematic analysis.

**Results:**

The contexts of the two health zones were similar and characterized by the existence of several community groups, similarly structured and using similar decision-making processes. They were not involved in the health sector’s activities and had no link with the health committee, even though they acknowledged its existence. They were not networked as they focused on their own activities and did not have enough capacity in terms of social mobilization or exerting pressure on public authorities or providers.

Women were not perceived as marginalized as they often occupied other positions in the community besides carrying out domestic tasks and participated in community groups. However, they were still subject to the local male dominance culture, which restrains their involvement in decision-making, as they tend to be less educated, unemployed and suffer from a lack of resources or specific skills.

The socio-economic context is characterized by subsistence activities and a low employment rate, which limits the community members’ incomes and increases their dependence on external support.

The governance context was characterized by imperfect implementation of political decentralization. Community groups advocating community rights are identified as “political” and are not welcomed. The community groups seemed not to be interested in the health center’s information and had no access to media as it is non-existent.

**Conclusions:**

The local contexts in the two health zones seemed not to be supportive of the operation of social accountability initiatives. However, they offer starting points for social accountability initiatives if better use is made of existing contextual factors, for instance by making community groups work together and improving their capacities in terms of knowledge and information.

**Electronic supplementary material:**

The online version of this article (doi:10.1186/s12913-016-1895-3) contains supplementary material, which is available to authorized users.

## Background

Maternal mortality remains a major public health issue in developing countries including the Democratic Republic of the Congo (DRC) [1], a country classified among fragile and conflict-affected states [2, 3]. Current estimations place the maternal mortality ratio (MMR) in DRC at about 846 maternal deaths per 100,000 live births [4]. Nearly two-thirds of this is due to direct obstetrical complications including hemorrhage, eclampsia, sepsis, obstructed labor, and unsafe abortion [5, 6]. The remaining one-third is due to indirect causes or pre-existing medical conditions made worse by pregnancy or delivery such as malaria, anemia, hepatitis, HIV-AIDS, tuberculosis, and malnutrition [6, 7]. Pregnancies are also occurring too early, too close, too late or too many times as suggested by the high fecundity (6.6 children per woman), early fecundity among adolescents (21.2 %) and the short inter-genesic interval (27.1 % births) [4]. Other factors associated with maternal mortality involve health systems weaknesses [8, 9], including the poor availability of reproductive health goods and services [10], socio-cultural barriers [7, 11], and armed conflicts [7, 12].

To address this high maternal mortality, DRC subscribed to the recommendations of the Safe Motherhood Initiative (SMI) [6, 13–16], International Conference on Population and Development, and the fifth Millennium Development Goal (MDG 5). Implemented SMI interventions led to a real improvement of maternal health indicators [7, 17–19] but still fell short of the 2015 MDG 5’s target [1, 4]. The low achievement of SMI targets has led some authors to call for significant efforts to improve and expand existing survival measures known to stem maternal deaths. They have encouraged setting up additional measures such as taking into consideration the perception of women [6] and removing financial barriers [6, 20, 21]. They also propose integrating global health priorities interventions, for example HIV services and antenatal care provision [6, 22]. Other authors suggested adding new interventions that target the providers’ behavior and responsiveness [13, 14, 23–25] in line with social accountability initiatives [26, 27].

Social accountability is defined as “accountability that relies on civic engagement i.e. in which citizens and/or civil society organizations participate directly or indirectly in exacting accountability” [28] and holding politicians, policy makers and healthcare providers responsible for their performance [28–31]. According to Lodenstein et al. (2013), social accountability comprises two main components. The first is citizen engagement, which includes individual participation in service provision and expressing one’s expectations and concerns in an effort to influence government policy, governance processes or other public services such as health services (voice). The second is citizen oversight, which includes involving citizens in the collective monitoring and evaluation of health services and the performance of health service providers, sanctioning when poor performance occurs and rewarding when the performance is perceived as being good [32].

In DRC, as in many developing countries, the beneficiary population is involved in health services including maternal healthcare through community participation. Community participation is one of the pillars of the national health policy, based on primary health care strategies [33]. In general, the rationale for community participation in health is to better respond to communities’ needs, designing programs that take into account contextual influences on health, and increasing public accountability for health [33]. In this article, we draw references to social accountability as one form of community participation and as discussed in literature [28, 32, 33].

As part of community participation, social accountability is viewed as a process of empowerment and as a social practice, in which communities are actively involved in changing the conditions that affect their health. Several authors such as Bukenya et al. (2012), Lodenstein et al. (2013), and Joshi (2014) argue that social accountability interventions and their effects are influenced by contextual factors, such as societal values, gender relations, levels of political stability and health system characteristics [32, 34, 35]. According to Thindwa et al. (2003), these contextual factors can assist or hinder the community, individuals or groups in promoting the community’s interests [36]. This indicates a need to understand the various local settings that can support or hinder the implementation and outcome of a social accountability intervention that aims to improve maternal health services.

This paper aimed to answer the following research question: What existing local contextual factors can influence the shaping, the implementation or the running of a social accountability initiative and the capacity of the community members, specifically women, to be engaged in it?

## Methods

A multiple case-study approach was employed to identify local contextual factors and discuss their possible influences on shaping, implementing and running social accountability initiatives at local level using qualitative research methods. It was conducted from May to June 2013 in two health zones (HZ) of DRC, the Muanda HZ (Kongo Central) and the Bolenge HZ (Equateur). These HZ were purposively selected. The case study inclusion criteria were: 1) health zone in post-conflict situation currently involved in sustainable development activities; and 2) the presence of health sector partners implementing or planning to implement health interventions including social accountability components for more than 4 years, targeting amongst others the improvement of maternal health. Details of the selected HZ are described in Table 1.

**Table 1:** Essential contexts indicators of selected study health zones

Indicators	Muanda	Bolenge
Province	Kongo Central	Equateur
Location in DRC	Southwest	North central west
Population	137 178	79 648
Number of health centers	9	15
Number of referral health facilities	2	1
Health facility attendance rate (%)	43.8	46.5
Antenatal health care attendance rate	98.0	91.3
Proportion of pregnant women with more 4 visits and more	46.2	40.2
Health providers’ attendance at birth rate (%)	95.1	78.4
Main population occupations	Agriculture	Agriculture
	Fishery	Fishery
	Small trade	Small trade
	Oil Firm employment	
Population composition	Bantu ethnic groups	Bantu ethnic groups
		Pygmies
Existence of other basic services		
Safe water supply	Yes	No
Electric power supply	Yes	No
Benefitting from a health intervention with social accountability components	Yes	No

An initial exploratory discussion were held separately with HZ officers and main community leaders to map out key community actors involved in maternal health at the local level, from which a representative sample was purposively selected to participate in the interviews. Among these community actors included public officers such as health services providers, political and administrative authorities, HZ authorities, and community representatives such as community leaders, community group members, women groups members, health committee members, and community health workers. The project managers of the NGO projects in both HZ were also included in the sample. Participants were purposively selected using maximum variation and identified from the pool of actors listed above. Selection was based on gender, age, involvement at community level activities in relation to health or other administrative functions. The selected individual were then approached through community health workers (CHWs) or HZ officers in-charge of community activities to participate in the interviews. No contacted individual refused to participate.

The interview guides were based on a conceptual model built on the framework and key concepts from Thindwa et al. (2003) enriched by those drawn from Marston et al. [33], McCoy et al. [37], Bukenya et al. [34], and Lodenstein et al. [32]. The framework from Thindwa et al. distinguishes four contextual factors that can enable or constrain the capacity of community members to engage in community development activities at the national and local levels in a sustained and effective manner. These factors are “the legal and regulatory framework; the political and governance context; socio-cultural characteristics; and economic conditions”. They in turn influence the “enabling elements” which are: “the freedom of citizens to associate (Association); their ability to mobilize resources to fulfill the objectives of their organizations (Resources); their ability to voice i.e. formulate, articulate and convey opinion collectively (Voice); their access to information, necessary for their ability to exercise voice, engage in negotiation and gain access to resources (Information); and the existence of spaces and rules of engagement for negotiation and public debate” (Negotiation). In this study, we put together the legal and regulatory framework with the political and governance context, and we extend the concept of resources beyond financial ones. We used this framework to explore if the context in the selected districts in DRC is enabling the shaping and implementation of social accountability interventions/mechanisms. Some variables related to community participation drawn from Marston et al. [33], McCoy et al. [37], Bukenya et al. [34], and Lodenstein et al. [32] were used to further operationalize the main factors in the framework, such as societal values, status of women, health committee recognition by the community and its interface role. The interview guides were adapted, pretested, and validated for the DRC local settings and for maternal health by the study team (see Table 2).

**Table 2:** Local contextual factors analysis conceptual model

Enabling elements	Socio-cultural characteristics	Legal and regulatory framework and Governance context	Socio-economic conditions
Association	- Existence of social structures supportive of community participation - Existence of actors involved in maternal health issues in local settings - Existence of community network, organizations or groups - Existing local experience of participation or of citizen engagement - Women’s status /Gender barriers - Level of women participation in communities’ activities	- Existing political system - Existing of national/local political context supportive of community participation - Freedom of association - Existing recognition and accreditation policies and practices related to the freedom of association, of information, of convening meetings	- Socio-economic characteristics of population - Impact of local economy on members’ contribution, on association autonomy and advocacy - Impact on contribution by members and cost of convening meetings - Cost of legal registrations and accreditation
Resources	- Social mobilization capacity within the community - Co-memberships - Existence of a history of community mobilization or social/citizen engagement - History of interactions between associations/groups - Decision making process within groups	- The individual capacity to collective action (social mobilization) - Decentralization	- Availability of basic services such as water supply, electricity/Infrastructures - Main occupations of the population/Earning potential of the population/ Size of and stresses in the economy unemployment
Voice	- Existing media/Access to media/ Communication practices in local settings (use of media by different social groups)	- Level of political control of means of expression/media - Freedom of expression - Media related laws	- Cost associated with expressing views in media
Information	- Access to Information - Information network - Literacy	- Freedom of information - Rights to access public information/Ability to demystify information	- Cost for access to information
Negotiation	- Existing social values and hierarchies - Distribution of ethnicity and tribes - Existence of excluded or marginalized population/social inclusion - Existing social structures in place that enable women to actively participate - Social capital/social pressures capacity/capacity of actors or groups to negotiate change - Level of women’s participation in decision making	- Existence of legally established dialogue spaces such as referendum or forum in local level/Existence of health committee - Level of trust officials have in the demand or the organization mobilizing citizen action - Local government authorities’ capacities to engage	- Bargaining power - Impact of economic constraints in autonomy and advocacy

Data were collected through individual semi-structured interviews and a document review. At each study site the research team interviewed selected actors. Face-to-face interviews were held in a quiet place away from other people to optimize privacy, and lasted 35 min on average. They were conducted in French or Lingala, and tape-recorded with the participants’ permission. There were no follow-up interviews as these were single-round interview discussions.

A documentary review was used to collect information on the health center’s activities, community groups’ activities, and socio-economic, political, and demographic data using a data collection form. Documents reviewed included the health center’s annual reports, health projects’ annual reports, health committee’s monthly reports, and some national policy documents.

Recorded in-depth interviews were transcribed verbatim. The interviewers proofread the transcribed work to cross-check accuracy of content since the interview transcripts were not returned for participant check and comment. The interview transcripts and data extracted from the documents were analyzed using the thematic approach [38], based on our context analysis conceptual model. A coding plan was developed using data from the first three interview transcripts and the core concepts of the conceptual model. Two members of the research team read and re-read each transcript thoroughly and assigned codes to each section of the text. Data processing was performed using Atlas-ti 6.1.1© software (ATLAS-ti GmbH, Berlin). Thematic analysis was performed to build a common and comprehensive understanding of the local context with respect to themes expressed by community members, triangulated by those coming from providers and public officers and the document review. Four steps were taken to enhance the credibility of the study: the research team received training in interview techniques, the interview guides were pre-tested and adapted accordingly; the results and interpretations were critically discussed by the research team and shared with local health partners and participants. The interview guides were written in French, translated into Lingala, and translated back into French.

## Results

The section starts with a description of the participants followed by three sub-sections presenting research results according to the specific contexts: socio-cultural, governance and socio-economic. In this presentation, findings from the two sites are presented together if they are similar, and separately when they differ between the sites.

In total, 35 semi-structured interviews were conducted with participants in the two sites. Table 3 presents an overview of the participants according to the type of their organization and expertise.

**Table 3:** Participants according to type of organization and expertise

	Expertise	Location		Sex		
Categories		Bolenge	Muanda	Male	female	Total
Community health workers/Health committee	Community participation, local organization	3	2	4	1	5
Community groups	Community organization, community development activities, networks, social activities, health-related problem support	6	7	7	6	13
Community leaders and traditional authorities	Community knowledge, social organization	2	1	3	0	3
Health partner	Local health service development, health interventions, community-based organization support,	1	1	2	0	2
Health providers	Health center management, community participation supervision, health provision	2	1	2	1	3
Health zone management officers	Health service organization and management, community participation, health service supervision	2	3	5	0	5
Local public administration officers	Community administration, local development	1	1	2	0	2
Total		17	16	25	8	33

## Socio-cultural characteristics

### Existing community associations and groups

The interviews revealed that the communities in the two sites have several formal and informal community organizations, associations, and groups of varying sizes, hereafter referred to as community groups. While their exact number is not known, they all have a similar structure: an executive committee supported by a general assembly. Almost all of the community groups have statutory documents such as internal regulations and statutes, which guide the group’s decision-making process. However, only formal community groups have submitted their statutory documents to the local administrative office for authorization. Decision-making within existing community groups takes place through meetings and the plenary assembly. Most of them asserted that by collecting their members’ expectations or views, which were discussed and debated during meetings, the final decisions were made by consensus. The interviews revealed that community groups did not take into account the views of non-members.

Apart from the health committee, all other community groups have a special focus and can be broadly classified into five main categories: (i) *Financial support groups* such as local mutual aid associations (LMAA), groups for mutual financial/professional support called “ristourne”, and community health insurance schemes; (ii) *Faith-based groups* such as faith-based youth’s or women’s associations and churches; (iii) *Collective or common-interest groups* such as associations of vulnerable persons, youth associations, women’s associations; (iv) *Groups working on local development issues*: nongovernmental organizations (NGO), community groups for development; and (v) *Activity-based groups* such as dialogue structures with firms, cooperatives, village committees, groups advocating the right of natives/professional groups. Brief descriptions of the main community groups obtained from the interviews are provided in Additional file 1.

**Fig. 1:**
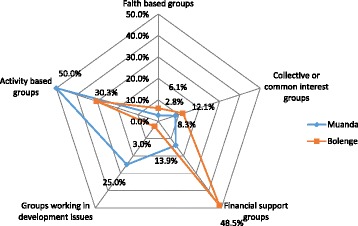
Distribution of community groups in % of references made by participants in the two sites

These community groups were distributed differently across the research sites. Not all participants were aware of the existence of every community group. We used radar charts to indicate the percentage of specific community groups that were mentioned by participants (Fig. 1). In the chart, community groups which have similar goals are grouped together. In Bolenge, the most frequently mentioned community groups were financial support groups (48.5 %) and activity-based groups (30.3 %). In Muanda, the most frequently mentioned ones were activity-based groups (50.0 %) followed by groups working on development issues (25.0 %).

The study allowed us to compile a list of actors (individual or groups) that could be involved in maternal health according to the participants’ opinions. The majority of the participants at both sites mentioned the community groups’ leaders, apart from health providers and members of the health committee. An interesting finding is that users were mentioned as actors in Bolenge but not in Muanda.

### Experiences in social mobilization and networking

Many participants from community groups within the two health zones declared that their groups had participated directly or indirectly in solving community problems, although there were few perceived social mobilization activities within the community. Existing community groups seemed to be focused only on their core activities and rarely extended their activities to mobilize citizen and state actors to engage in community activities or extended their activities to the health sector. Most community group members explained that their groups were not involved in the health sector’s activities and had never taken decisions concerning health nor public health service provision. They stated that the community members’ engagement in the health sector was organized around the health committee.

However, some past experiences of participation in community activities by these groups exist at both sites: for example, by supporting and mobilizing their members to contribute to the building of the local school. Two types of community groups, those working on local development issues and activity-based groups, seemed to manifest more social mobilization and advocacy for action than other community groups. They had both benefitted from technical/financial support and capacity building provided by external partners such as international NGOs and enterprises, which were more present in Muanda. Nevertheless, some participants (health providers and public officers) asserted that those social mobilization activities were rarely initiated by the community members themselves but were organized and piloted by external organizations.

With respect to networking, it is apparent from the data that very few relationships were established between the local groups themselves, between the local groups and external NGOs, and between the local groups and governmental bodies. There was no shared networking platform between the groups, and they did not conduct joint activities.

Furthermore, some participants pointed out that the community groups did not have enough capacity and expertise to express their views or to exert pressure on the public authorities or health providers. A few participants, mainly the community group representatives, thought the opposite, asserting that it was the ineffectiveness or the lack of responsiveness from health providers and public authorities which dissuaded them and made them less pro-active. Other participants argued that they lacked a champion to take their expectations and needs to the health providers/public authorities. Some community group representatives explained that the community groups’ capacity to express views or exert pressure was also hindered by their inability to build cross-boundary alliances or coalition with other groups, as local authorities use the strategy to individualize the population’s demands or dismantle the most active groups.

*“Local associations did not sufficiently manifest their capacity to be the voice of the community in front of authorities or other persons. We think that the community voicing does not function and authorities would not response to our request. We had not yet identified a community group that could speak up and influence the decision-making.”*

*(*Male, community group member)

However, several community group representatives stressed that some groups working on local development issues and some activity-based groups in Muanda had benefitted from training and were currently contracted by the Muanda Funds Holding Agency, a partner of Cordaid, to monitor the health center’s performances through a community verification survey.

#### Cultural diversity and marginalized population

Both health zones house a large number of tribes and ethnic groups (more than 10). Participants perceived that this multiplicity of tribes and ethnic groups did not constitute a problem for the constitution and functioning of community groups.

*“Our village comprises inhabitants that came from other tribes such as …The cultural identity and customs of each person do not affect the functioning of our community groups. This large variety of cultures does not influence the function of our groups.”*

(Male, community group member)

Nevertheless, health project managers and health providers revealed that sometimes friction occurred between natives and non-natives, particularly in affluent locations such as Muanda.

*“Here, we sometimes have some problems. Natives are …and did use to call other people foreigners and sometimes marginalize them.”*

(Female, community group member)

When asked about marginalized groups, most people mentioned the Pygmies in Bolenge and Basolongo in Muanda. Participants asserted that members of those groups were less integrated with other community groups, had their own social system, were generally more vulnerable, less educated, and poorer, and had less access to employment. According to a public officer, the government made efforts in terms of sensitization and education to reduce marginalization and increase their integration with other groups. Despite these efforts, some community group members explained that people from these marginalized groups did not become members of existing community groups for financial, religious, or personal reasons.

### Women’s status and participation in community groups’ activities

With respect to women’s status, many participants asserted that women were not marginalized in the community, arguing that women often occupy important positions in the communities and are not solely consigned to domestic tasks.

*“The women participate in management or within the associations, a woman can be president, vice president, advisors and men are members as well.”*

*(Male, community group member)*

*“In the community, it is true that before women were less considered than men. However, nowadays with the action of non-governmental organizations, the effort of the state through the education of women, they are equal. A woman can realize what she wants depending on competences and skills she has. For example, the in-charge of the health center is a lady…”*

(*Female, community group member)*

The interviews revealed that women participated in the community groups. With respect to their composition, the local communities have groups with only women or only men as members, and others that included both men and women. In the latter category, women participated in decision-making during the general assembly and plenary and were also elected to governing bodies. However, most of the time, women were appointed to positions such as treasurer, social assistant, caregiver or group’s advisor, which, according to some participants are associated with the traditional view that women have a higher caring capacity and sense of righteousness and honesty.

Nevertheless, a few participants stated that there are differences between men and women. A health provider from Bolenge, for example, asserted that women did not effectively participate in decision-making in the local society, linking this situation to the local culture of male dominance. As an example, this health provider stated that women more often come to the health center accompanied by their husbands or their mothers-in-law and were rarely the chairperson in groups that included both men and women.

*“Women are not really involved in decision making. It is the culture. Very often, when they are sick, they are always accompanied by them husbands when coming to the hospital. But I am not informed with regard to the decision-making within the associations where the women and the men are members”*

(*Female, Health provider)*

Very few participants argued that a woman could only be more active and autonomous in their local community if she had led a business with financial resources, possessed specific skills and competencies, occupied a political or economic position in society, or was well educated. However, most participants recognized that women at the local level rarely satisfied the above-mentioned conditions.

*“In reality, men and women are equal. The issue is that women in our environment…do not have the required competences or educational level for being effectively involved in decision-making.”*

*(Male, community group member)*

Some participants associated the perception of the improvement of women’s status with some community groups’ activities such as local NGOs that focused on women’s empowerment, and with the national education policy that encourages the education of girls, at least to the primary school level. They also mentioned some barriers to women’s empowerment and education such as the challenging socio-economic situations which prevent families from schooling their children, especially girls, and local customs which encourage early marriage.

*“Yes, women are very important, women here often work in fishery, the land/field or trading, thus they don’t study. We don’t really have women capable of working or expressing themselves very well. Often when women go to …there, that is all, they get married there and they have their life there. Here, women are not emancipated, maybe less than 20 % of them work, mainly as small traders.”*

*(Female, Women’s community organization)*

### Existing media and access to information

The interviews also revealed that media which could enable a large number of community members to be reached were relatively non-existent at the community level. Neither papers nor radio broadcasting or television were found at the local level. Even if some inhabitants could organize radio reception from a city situated in the neighborhood (more than 15 km away), these stations rarely broadcast local information. Health-related information exchanges are mainly based on interpersonal communication and sensitization, conducted by community health workers or in small-scale health education meetings organized in the health center. Except for members of the health committees, community members did not have access to information about the health center’s activities and asserted a lack of interest because they did not work at the health center. Alternatively, they thought that health providers would not appreciate their interest.

## Governance context

The two research sites are both located in health zones, which are part of the territory’s administrative system. Moreover, though the Congolese constitution prescribes the implementation of decentralization, several participants asserted that decentralization is not effective and local political entities have not yet been installed, such as the local councils and local elected representatives necessary for local political participation. They stated that the power and decision-making are still centralized at the national level or at the provincial level, and they expected more from the decentralization, such as the facilitation of administrative procedures and resource allocation.

*“Nothing more has changed with respect to the decentralization, and it is not effective yet.”*

*(Male, community group member)*

Despite this, most participants asserted that the political situation does not prevent the organization of interest groups. Some participants stated that the local political context sometimes favors group formation, even though community groups have to follow certain regulations and require authorization from the local political authorities, in order to hold public meetings and implement their activities.

*“The political level currently does not cause a problem for community groups. Existing groups have to respect the law. They must make themselves known to the political authority and follow the political and administrative regulations… by paying taxes and charges prescribed by the law.”*

*(Male, public officer)*

Some participants revealed that certain community groups, specifically those committed to human rights and community interests, are not welcomed by the authorities at the national or local level. They stated that the authorities readily considered a group committed to advocating the population’s interests, such as the right to health care or to education, as “political” because then it becomes the responsibility of the national government. These participants also added that the government or local authorities therefore considered that demanding one’s rights was equal to being critical of the government.

*“Community groups have to refrain from “bad” activities. If not, the state will intervene. The state can get involved if the groups address political matters, speak negatively of the government, or in case of public disturbances as well or open conflicts among members or in the community.”*

*(Male, public officer)*

This understanding of the commitment to human rights as a political activity induced several community groups to declare their apolitical nature and assert the freedom of their members from affiliation to any political party. Moreover, some participants (10/35) argued that the governance context could have a negative effect on the functioning of community groups especially during election times, as it drives the community groups away from their primary goals, patronizing them through donations and gifts to take a political position and to work for political parties.

*“The negative influence of the political context occurs during electoral propaganda, several people follow politicians who could give them money and gifts instead of getting involved in community engagement. They are sure to be beneficiaries of the generosity of politicians… The community participation disappears almost entirely during these periods of intense political activity*.”

*(Male, Health Zone management team)*

As an element of governance, some participants acknowledged that community activities associated with health were organized by the health committee, as required by the national health policy. They asserted that a health committee is composed of community health workers, whose members were responsible for community participation activities and acted as “bridges” between the community and the related health center. However, some participants claimed that the health committee was dependent on the health center team, which provides funding and directions for its activities. They also stated that most of the health committee members were not elected by the community but were chosen by the nurse in charge of the health center, and therefore, they concluded that the health committee members were not really representative of the community’s opinions.

With respect to the relationship between community groups and the health committee, participants stated that community groups did not have links to the health committee. However, they recognized that most of the health committee members and community health workers were also members of community groups, even though these groups did not seem to use this co-membership to develop links with the health committee or vice versa, and to be involved in health activities.

## Socio-economic conditions

There are some differences in the socio-economic conditions in the two sites. In Bolenge, there is neither a safe water supply nor electricity, and the majority of the population work in subsistence agriculture, fishing, or farming. The local wages are very low. The annual average per capita income is less than $298. The few people with a regular salary worked mainly as civil servants in the education or health sector with very low salaries [39]. The Muanda HZ, on the other hand, is a region with increasing oil production and profits from a strategic trading position between the borders of Angola and Congo Brazzaville. Muanda houses the agencies of several enterprises and banks. Firms which produced oil provided electricity and safe water to several villages and sometimes offered seasonal working opportunities to the local populations. These firms sometimes invested in local initiatives through the local development committee they established.

Despite these differences, the majority of community members at both sites are very poor. Most of them did not have enough financial resources to fund community activities and were inclined to believe that external partners always have funding to give them or to invest in their community projects or activities. Neither site received subsidies from the government.

## Discussion

The principal aim of this multiple case study were two-fold. The first aim was to identify local contextual factors in two DRC health zones. The second aim was to discuss their influence on the shaping, the implementation and the running of social accountability at the local level. To this end, we used a conceptual model adapted from Thindwa et al. [36] enriched by concepts drawn from Marston et al. [33], McCoy et al. [37], Bukenya et al. [34], and Lodenstein et al. [32] which allowed us to identify contextual factors that are necessary for community engagement and to match them with enabling elements for social accountability (Additional file 2).

This study has highlighted some enabling and constraining factors as being important in the shaping, the implementation and the running of a social accountability initiative at the local level.

### Enabling factors

This study shows that “Association” is facilitated by socio-cultural characteristics such as the existence of formal and informal community groups, the willingness of the population to support each other, previous positive experiences with community engagement, and the involvement of women in community groups. Governance factors which support “Association” at both sites include the existence of a regulatory framework for community groups, acceptance of community groups by the local authorities, and the national recognition of community health committees as legitimate health governance bodies. The socio-economic conditions in Bolenge motivated community members to form groups in order to pool their meager resources through mutual aid associations.

Potential capacities to mobilize “Resources” to fulfill their objectives exist in Bolenge and Muanda in terms of socio-cultural characteristics such as the co-membership of several community groups; a history of social mobilization activities by some community groups; and the use of discussion and debates for decision-making within community groups. In addition, in Muanda oil firms and NGOs supported some local community groups in the form of capacity building in organization and funding.

Regarding “Negotiation”, potential space and rules of community engagement exist as health committees are the legal interface between the community groups and the health providers, although currently these committees do not function optimally according to respondents.

### Constraining factors

Regarding “Association”, constraining socio-cultural characteristics include the lack of networks and platforms between groups. In addition, community groups seem to have a narrow focus on their own core activities and insufficient capacity for community mobilization.

Concerning “Resources”, socio-economic conditions of limited employment opportunities and meagre income from subsistence farming prevent community members from contributing to community projects. This situation, associated with a lack of government funding, makes community groups dependent on external financial support.

Regarding “Voice” and access to “Information”, limitations were found in the low coverage of radio and other media at rural levels. It was also observed that community members did not seem interested in information related to the health services’ performance. An underlying reason might be the low socio-economic conditions and corresponding low level of education among community members, especially women. An additional socio-cultural constraint is the weak capacity and expertise of community groups to express their views or to exert pressure on service providers.

Constraints in “Negotiation” play out in the governance context as local authorities hinder community groups that are promoting the interests of citizens. Additional “Negotiation” constraints include the selection of health committee members by public health providers rather than community members, and the neglected interface function of health committees. The authorities even employ active strategies to individualize the population’s demands and dismantle the most active community groups promoting community interests. In addition, decentralization is not fully implemented, and decision-making regarding the health services and other basic services takes place at the central level, which limits people’s influence on the decision-making and accountability of local authorities.

The situation described here seems unfavorable and presents limitations to shaping, implementing and carrying out social accountability for health service improvement.

Several authors underlined the importance of some contextual factors necessary to enable the harmonious implementation and smooth running of social accountability, which are lacking in these health zones, such as the existence of a coalition and social mobilization [26, 28, 33, 35, 40–44]. Other authors stressed the importance of the capacity of community groups to express their views or to exert pressure on health providers or on the public authorities [45, 46], a well-functioning health committee [45, 47] especially considering the limited influence of health providers [48, 49], competent decentralization [45, 50], and the role of the media in providing access to information [28] as enabling factors of social accountability.

Regarding constraints, several authors have also identified some contextual factors that can hinder social accountability such as the low status of women [26, 44, 51, 52] and the identification of social actions promoting citizens’ interests as a political activity.

However, the existing context in the two health zones in DRC could offer several starting points to initiate social accountability in local maternal health services [36]. Better use could be made of existing community groups for enabling the local context through strengthening coalition building among themselves and between them and the health committee in line with Falisse et al. (2012) in Burundi [48] and Dasgupta (2011) in India [26], and building capacity in terms of an interface role, of generating and using information about the health center’s performance, of knowledge/information about entitlements and the health service performance in line with experience provided in the social accountability literature [44, 46, 49, 53, 54].

To proceed, one option is to use the co-membership of some community members in the health committee and in existing community groups as an entry point for building coalitions, which is a process of negotiation, building interactions, and creating common trust among existing actors and groups [55]. One way of strengthening coalition-building is through the use of participative approaches, such as the interactive learning and action approach. For instance, Swaans et al. (2009) in South Africa, Björkman and Svensson (2009) in Uganda, and Dasgupta (2011) in India provide an overview of a coalition-building process around HIV and agriculture, community monitoring of health care and maternal health. In DRC the coalition could be built around the health committee and community health workers, as they are perceived by other community members as bridges between the community and the health providers with the support of the HZ management team and health partners. This coalition could support community mobilization strategies to enhance participation at the local level and strengthen existing community groups.

The second line of action is capacity-building. This can be done through the involvement of community groups using participatory approaches in generating information on their own views and concerns, in discussing them with health committee members and health providers, and by making information available to them [37, 56]. Capacity-building of community groups can also be done by involving them in community problem-solving. They would then be involved in defining, implementing, monitoring, and evaluating health activities. Some examples of successful capacity-building interventions in local settings using participative approaches are provided by Swaans et al.(2008) in South Africa [57], Björkman and Svensson (2009) in Uganda [53], Katahoire et al. (2015) in Kenya [58], and Manandhar et al. (2004) in Nepal [22].

The capacity-building would also concern the health committee. It can be used to support its interface role better [59], which is necessary to facilitate communication and dialogue between community members and health providers through training and supervision [45, 50]. The women should occupy a special place in these initiatives so as to improve their capacities to be pro-actively engaged in decision making and involvement in these health initiatives.

### Implication for policy

Contextual factors such as described in the present study are more likely to be somewhat found at the local level in several low-and middle income countries, as reported in existing literature from Benin [23, 51], Burundi [48], Uganda [58], Tanzania [60] and India [26, 44]. This highlights that the conceptual model as adapted can be used in other setting in order to generate information about local contexts. This information can be used to shape more appropriate actionable interventions with regard to social accountability.

### Study limitations

Generalization of our findings is limited because of its case study nature and the small number of health zones in which the study took place. In addition, we did not have data on all contextual factors provided by the conceptual model and did not explore the effects of national-level contextual factors on local settings [28]. However, the findings in our study are largely in line with the literature, and the participants’ responses were largely overlapping. The study thereby provides useful starting points for further research on contextual factors influencing the shaping, the implementation and the functioning of social accountability initiatives in local settings. Second this study is the first to provide in-depth insights of local level contextual factors [44]. Previous studies contributed more to national and subnational levels [26, 34–36, 59]. Third, the modified conceptual model could be used as an analytical tool in other context different from DRC.

### Research team and reflexivity

The researcher in charge of interviewing participants was a medical doctor, trained in maternal health practice and with a background in quantitative methodology. He and most of the respondents were of the same age. He introduced himself as a researcher from the local university. He noted his impressions after the interviews, and they were discussed during a daily debriefing meeting with his supervisors. His notes were included in the data analysis. The latter was mainly conducted by the first author and the second author, who has a social sciences background. Their backgrounds could have influenced the data analysis and interpretation. To reduce these influences, the data analysis was conducted using the framework and involved extensive interaction with supervisors. They read the narrative on their own terms, and judged how they were responding emotionally and intellectually to this person. They put themselves, their background, history, and experiences in relation to the respondent and inserted their findings into the framework. This allowed the authors to examine how their assumptions and views might affect their interpretation of the respondent’s words, or their writing about the person. Research team members had no relationship with participants prior to study commencement. Participants learned about researchers and the research during consent administration. All the stages of data analysis were supervised by three supervisors with experienced in qualitative data analysis, and the findings were discussed with a Social, Policy and Administration Sciences specialist from the University of Kinshasa, DRC.

## Conclusions

The local contexts in the two health zones seemed not to be supportive of the shaping and implementation of social accountability initiatives. However, they offer starting points for social accountability initiatives if better use is made of existing contextual enabling factors, for instance by making community groups work together and improving their capacities in terms of knowledge and information.

## Additional files

Additional file 1: Brief description of main community associations and groups as emerged from interviews. (DOCX 13 kb)
Additional file 2: Contextual Factors analysis conceptual model/Mapping of Data. (DOCX 14 kb)
Additional file 3: Interview guide translated in English. (DOCX 24 kb)

## Abbreviations

CHWs: Community Health Workers
DRC: Democratic Republic of the Congo
HZ: Health Zone
LMAA: Local mutual aid associations
MDG: Millennium Development Goal
MMR: Maternal mortality ratio
NGO: Nongovernmental organizations
SMI: Safe motherhood Initiative
