# Supplementary material for: Understanding the local context and its possible influences on shaping, implementing and running social accountability initiatives for maternal health services in rural Democratic Republic of the Congo: a contextual factor analysis
Source: BMC Health Serv Res. 2016 Nov 9;16:640. doi: 10.1186/s12913-016-1895-3 (PMC5103494; doi:10.1186/s12913-016-1895-3)
Supplement: Additional file 1: — Brief description of main community associations and groups as emerged from interviews. (DOCX 13 kb) [file 12913_2016_1895_MOESM1_ESM.docx]

**Additional file 1. Brief description of main community associations and groups as emerged from interviews**

1. **Local mutual aid association**

A local mutual aid association (LMAA) is a group at the level of a village that is based on religious, professional affinities or residential closeness, and collects financial contributions following a fixed frequency to support each other under certain conditions. It is composed of an executive committee and the members, who together constitute a general assembly. It is governed by an internal regulation order or statutes. Generally, executive committee members are not paid a salary, and the association does not offer paid lucrative services.

LMAAs are purely local, without any links to external partners. In certain cases, they form structures of dialogue, intended to manage the relations and the conflicts between them. Some are constituted only by women. LMAA can take several forms, such as women's associations, associations of natives, and farming women associations.

1. **Local development nongovernmental organization (NGO)**

The nongovernmental organizations engaged in development activities are groups of people around core activities. These people share certain competencies allowing them to realize their goals. In the contexts of DR Congo, NGOs often look for financing to implement activities. They are active in the fight against HIV AIDS, sexual violence, and poverty. Even if the members put together funds, the survival of these associations depends mainly on external financing. Most of them have benefitted from training provided by external partners like the United Nations Development Program (UNDP), Congo Competences or Cordaid to improve the functioning of their organization. Often, NGOs count fewer local members than LMAA associations.

1. **Associations advocating the interests of natives or professional groups**

Associations advocating the interest of natives and professional groups such as fisherman or farmers are groups of persons exercising the same profession or natives living in a region with natural resources which are exploited by companies or firms. They are constituted to advocate the interests of their members or help them to work together efficiently. They are especially numerous in Muanda Health Zone due to the oil exploitation and the fisheries. Their activities focus solely around their objectives, and their members are not interested in other sectors.

1. **Dialogue structures with firms**

The dialogue structure is mentioned mainly in the Muanda Health Zone. It is a group of community representatives, coming from different backgrounds, that works like an interface between firms that run mineral resources exploitation and the natives. It coordinates the population’s demands and is in charge of validating the small community projects such as building a dwelling pit or a school before submitting them to the partner companies for financing. The dialogue structure is often initiated by the firms themselves, which have a special administrative unit in charge of the local development affairs. Many members of dialogue structures have benefitted from training in local development and community participation.
