# Supplementary material for: Understanding the local context and its possible influences on shaping, implementing and running social accountability initiatives for maternal health services in rural Democratic Republic of the Congo: a contextual factor analysis
Source: BMC Health Serv Res. 2016 Nov 9;16:640. doi: 10.1186/s12913-016-1895-3 (PMC5103494; doi:10.1186/s12913-016-1895-3)
Supplement: Additional file 2: — Contextual Factors analysis conceptual model/Mapping of Data. (DOCX 14 kb) [file 12913_2016_1895_MOESM2_ESM.docx]

Additional file 2. Contextual Factors analysis conceptual model /Mapping of Data

| Enabling elements | Socio-cultural characteristics | Legal and regulatory framework and Governance context | Socio-economic conditions |
| --- | --- | --- | --- |
| Association | Existence of social structures supportive of community participation (+)  Existence of actors involved in maternal health issues in local settings (+)  Existence of community network, organizations or groups (-)  Existing local experience of participation or of citizen engagement (+)  Women’s status /Gender barriers (-)  Level of women participation in communities’ activities (+) | Existing political system (+)  Existing of national/local political context supportive of community participation (+)  Freedom of association (+)  Existing recognition and accreditation policies and practices related to the freedom of association, of information, of convening meetings (+) | Socio-economic characteristics of population (-)  Impact of local economy on members’ contribution, on association autonomy and advocacy (±)  Impact on contribution by members and cost of convening meetings (-)  Cost of legal registrations and accreditation (-) |
| Resources | Social mobilization capacity within the community (-)  Co-memberships (+)  Existence of a history of community mobilization or social/citizen engagement (+)  History of interactions between associations/groups (-)  Decision making process within groups (+) | The individual capacity to collective action (social mobilization)(-)  Decentralization | Availability of basic services such as water supply, electricity/Infrastructures (±)  Main occupations of the population/Earning potential of the population/ Size of and stresses in the economy unemployment (-) |
| Voice | Existing media/Access to media/ Communication practices in local settings (use of media by different social groups)(-) | Level of political control of means of expression/media  Freedom of expression  Media related laws | Cost associated with expressing views in media |
| Information | Access to Information (-)  Information network  Literacy (-) | Freedom of information  Rights to access public information/Ability to demystify information | Cost for access to information |
| Negotiation | Existing social values and hierarchies (-)  Distribution of ethnicity and tribes (-)  Existence of excluded or marginalized population /social inclusion (-)  Existing social structures in place that enable women to actively participate (+)  Social capital/social pressures capacity/capacity of actors or groups to negotiate change (-)  Level of women’s participation in decision making (-) | Existence of legally established dialogue spaces such as referendum or forum in local level (-)/Existence of health committee (+)  Level of trust officials have in the demand or the organization mobilizing citizen action (-)  Local government authorities’ capacities to engage (-) | Bargaining power (±)  Impact of economic constraints in autonomy and advocacy (±) |

Legends: (+): enabling factors (-): Constraining factors No sign: no observed factor
