# Supplementary material for: Understanding the local context and its possible influences on shaping, implementing and running social accountability initiatives for maternal health services in rural Democratic Republic of the Congo: a contextual factor analysis
Source: BMC Health Serv Res. 2016 Nov 9;16:640. doi: 10.1186/s12913-016-1895-3 (PMC5103494; doi:10.1186/s12913-016-1895-3)
Supplement: Additional file 3: — Interview guide translated in English. (DOCX 24 kb) [file 12913_2016_1895_MOESM3_ESM.docx]

**KINSHASA SCHOOL OF PUBLIC HEALTH**

**WOTRO IMPROVING MATERNAL HEALTH THROUGH SOCIAL ACCOUNTABILITY IN DRC**

**INTERVIEW GUIDE WITH KEYS INFORMANTS**

**HEALTH ZONE: ……………………… HEALTH AREA: ……………………… DATE: ………………………………………**

**A. Introduction**

The Government of the Democratic Republic of the Congo gets support from other governments from other countries and organizations to fund projects in the health sector. All these projects aim at improving health situation of the population and ensuring that the population have access to quality and comprehensive health care and effectively use the available health services

Among his missions, the Kinshasa School of Public Health aims at supporting the Ministry of Public Health by collecting data in health facilities and communities in order to provide relevant information for decision making. This interview is set in this context. It is organized to discuss with representatives of the community and other stakeholders about health problems, support they perceived and solutions provided.

**B. Informed Consent process *(to be declared to the interviewee)***

My name is ………………………….I am working at Kinshasa School of Public Health, which is a part of University of Kinshasa. I would like to discuss with you and ask you some questions about your community and related health problems. The aim of this interview is to understand how the community is organized in the health zone with regard to health and how different organizations, projects, persons involved in the health sector are working at local level. The information that will be collected will be used to inform the Ministry of Public Health in how to better organize the health provision. I would like to use a voice-recorder during the conversation for better capturing your opinions and would like to have your permission to use it during the discussion. I will show you how it is functioning so you can stop recording when you do not feel comfortable. I will also sometimes take notes in a notebook about the discussion in order to follow up with additional questions.

All discussion will turn around the local community and around health issues. The discussion and your opinion will be kept confidential and will be used only in the purpose of this study. The notes taken during the interview and all materials will be saved in secured places. The recordings will be transcribed verbatim using a computer and afterwards destroyed. During interview, your name or your position will not be mentioned. Some of your personal information will be collected at the end of the interview and written out in the transcript of this interview in a manner to keep anonymous and confidential your identity.

Other persons involved in this project such as supervisors will have access to the transcripts. Additionally, data from this research will be published in review or shared with other persons. In this case, all required strategies will be used in order to ensure the anonymity. All persons involved in the research project are subject to professional ethics and are committed themselves to ethical considerations. Publications that can be produced from the data will all adhere to ethical issues. Your participation is free, you can stop or take decision to not answer to a question without risk for you. You will not receive any direct benefit from participating in this study but information that will be collected will be used to inform better health decision and policy.

You can ask question or make comments on the study and if you have more question or concerns regarding this study and your participation, you can call [Name]………………………………………………….in charge of this research using this phone number…………………………………..or the president of Kinshasa School of Public Health ethics committee.

Name……………………………………………. Phone number………………………………………………………

Do you agree to this? ……………………………………………

You can sign here in order to testify that you were informed and provide you consent:

…………………………………….

Can I begin?

**C. Guide 1. Interview guide for Key informants**

| **N°** | **Questions (Analyse de context)** |
| --- | --- |
|  | **INTRODUCTION**  We would discuss during the interview about the local context of your community. We will discuss about health problems, health organizations and support this community receives. We will also discuss how the community is organized and what its main features in terms of socio-cultural, political, administrative aspects are. |
| 100 | Would you like to present yourself without mentioning your name?  If you are representative of a community group or organization, could you briefly present it? (Probe for goals, missions, activities, achievements, target groups…) |
| 101 | In general, what are health problems population of this community face? Probe for Health issues, health services issues, health related issues?  What are the most concerned people?  Could you provide me what do you think are health needs of this community? |
|  | **HEALTH SUPPORT INTERVENTIONS** |
| 102 | In this health zone, could you provide me what organizations are working in the health sector? Could you link them to health problems you previously mentioned? |
| 102b | You have mentioned ………………………………. What activities do this organization implement in your community or in this health zones? |
| 103 | What are population needs targeted through the implemented activities?  Could you extend on the relevance of the implemented activities?  What results or benefits do you perceive from the implemented activities?  Could you provide some examples? |
|  | **ORGANIZATION OF THE SOCIETY** |
| 104 | What ethnic groups (tribal groups) live in this health zone?  How are this groups organized? Probe for community groups, political groups…)  How do these groups function? (Probe for each type of groups mentioned by the respondent)  How do you think that the existing community groups are involved in the implementation of health activities?  How do you think that the decision making is performed in this group? |
|  | **PLACE DE LA FEMME** |
| 105 | What is the place of women in your community?  What responsibilities are ensured by a woman?  How does woman participate in decision making in the community?  What could you say on community groups gathering women in your community?  What are strengths and weaknesses of this type of groups?  What are health activities that target women?  What are organization that work with women in your community?  What benefits do you think women have in working in community groups? |
|  | **POPULATION AND ORGANIZATIONS** |
| 106 | What organizations, associations or groups could have influence in the health sector or health problems in terms of power, capacity of pressure or community actions?  What sub-groups exist in the community?  How are those groups and sub groups involved in existing health projects and programs?  What role do they play in these projects?  How are they involved?  What interest do you think they have in the activities of other organizations involved in health sector?  How could these groups influence the local community and the health sector? What do you think can be their strengths, weakness or capacity to participate in health sector activities?  What collaboration do you get between existing groups?  What conflicts of interest do you perceived between them?  How do these groups communicate between them and between them and the community? |
| 105 | Could you provide for each of these groups what you think be its influence in existing projects? |
| 106 | What benefits do you think these groups can have from existing projects in health sector? |
| 107 | What contact do these groups have outside the health zone? |
|  | **ACCOUNTABILITY** |
| 108 | How do community group/associations get information about needs, concerns and expectations of their members? For people out of their groups?  How do people who are not members of community association/groups to make their voice heard by associations? |
|  | **POLITIC AND COMMUNITY PARTICIPATION** |
| 109 | What is your opinion about the current policy and political situation with regard to community participation? How does the current political situation influence community engagement and community groups?  What elements from the constitution or other policy do you think support the involvement of community in the management of their community?  What elements from the constitution or other policy do you think support the involvement of community in the management of their health or health services?  What elements do you think prevent community participation?  What elements do you think prevent community participation in the management of their community?  What elements do you think prevent community participation in health and in health services?  What elements do you think prevent social changes in your community? |
| 109a | What community mobilization activities are currently implemented in the community? Could you explain more these activities?  Who do you think take initiative of these activities? What support do organization receive for carrying out these activities (Probe for financial, technical, other…?) |
| **N°** | **INSTITUTIONAL AND LEGAL FRAMEWORK AND GOVERNANCE** |
| 110 | How do you think that political situation affect health sector?  How do you think that political situation affect community groups functioning?  What could you said about decentralization process and about decentralized political and administrative entities? |
| 111 | **FINANCING**  How do you think about socio-economic situation in your area? (Probe for employment, assets, earning potential, occupation, main activities…)  How do this socio-economic situation affect the functioning of local group?  How do this socio-economic situation affect the place of women in your community?  How do this socio-economic situation affect health services and health projects? |
| 112 | **SOCIO-CULTURELS ASPECTS**  Could you provide me some features regarding socio-cultural aspects in your community?  How do you think these features affect community participation?  How do you think these features affect the functioning of community groups?  How the community is informed about what is happened within it? Are there some media that can broadcast community problems? |
| 113 | **COALITION BUILDING**:  How do you think local groups manage the relationship among them?  What platform exist among local groups?  How do the community groups manage their relationship with the health sector at local level? |
| 114 | What relationships do community groups have with administrative and political entities?  What relationships do community groups have with political parties?  What relationships do community groups have with nongovernmental organizations and civil society? |
| 115 | What external actors do you know are interested in the local community?  How do these external actors perceive health projects and interventions with are implemented within the community? |
| 116 | What is your opinion about the organization health projects implemented within the community? |
| 117 | **SOCIAL ACCOUNTABILITY IN HEALTH PROJECTS :**  How do you think health project do for having needs, concerns, demands and expectation of their target groups?  Do you think that health projects know information about needs, concerns, demands and expectations for local community groups? If yes, could you reflect on? |
| 118 | What relationships exist between health projects implemented in the community and their target groups?  What is your opinion on this relationships? |
| 119 | **SATISFACTION AND PERCEPTION :**  As member of this community, how do you think that target groups assess services provided to them through the project? What is your opinion in their satisfaction? |

1. **Identification of respondent Number audio file  :**

***Instructions***: to be filled after the interview

| **Organisation** | **Occupation/Position** | **Sex** | **Age** | **Duration in the position** |
| --- | --- | --- | --- | --- |
|  |  |  |  |  |
